# TF-Cluster: A pipeline for identifying functionally coordinated transcription factors via network decomposition of the shared coexpression connectivity matrix (SCCM)

**DOI:** 10.1186/1752-0509-5-53

**Published:** 2011-04-15

**Authors:** Jeff Nie, Ron Stewart, Hang Zhang, James A Thomson, Fang Ruan, Xiaoqi Cui, Hairong Wei

**Affiliations:** 1 Morgridge Institute for Research, 330 N. Orchard St., Madison, WI 53715, USA; 2 Department of Biostatistics and Medical Informatics, University of Wisconsin, 600 Highland Ave., Madison, WI 53792, USA; 3 Department of Cell & Regenerative Biology, University of Wisconsin, 1300 University Ave., Madison, WI 53705, USA; 4 School of Forest Resources and Environmental Science, Michigan Technological University, 1400 Townsend Drive, Houghton, MI 49931, USA; 5 Department of Mathematics, Michigan Technological University, 1400 Townsend Drive, Houghton, MI 49931, USA; 6 Department of Computer Science, Michigan Technological University, 1400 Townsend Drive, Houghton, MI 49931, USA; 7 Program of Computing Science and Engineering, Michigan Technological University, 1400 Townsend Drive, Houghton, MI 49931, USA; 8 Biotechnology Research Center, Michigan Technological University, 1400 Townsend Drive, Houghton, MI 49931, USA; 9 Department of Molecular, Cellular, & Developmental Biology, University of California Santa Barbara, Santa Barbara, CA, 93106, USA

## Abstract

**Background:**

Identifying the key transcription factors (TFs) controlling a biological process is the first step toward a better understanding of underpinning regulatory mechanisms. However, due to the involvement of a large number of genes and complex interactions in gene regulatory networks, identifying TFs involved in a biological process remains particularly difficult. The challenges include: (1) Most eukaryotic genomes encode thousands of TFs, which are organized in gene families of various sizes and in many cases with poor sequence conservation, making it difficult to recognize TFs for a biological process; (2) Transcription usually involves several hundred genes that generate a combination of intrinsic noise from upstream signaling networks and lead to fluctuations in transcription; (3) A TF can function in different cell types or developmental stages. Currently, the methods available for identifying TFs involved in biological processes are still very scarce, and the development of novel, more powerful methods is desperately needed.

**Results:**

We developed a computational pipeline called TF-Cluster for identifying functionally coordinated TFs in two steps: (1) Construction of a shared coexpression connectivity matrix (SCCM), in which each entry represents the number of shared coexpressed genes between two TFs. This sparse and symmetric matrix embodies a new concept of coexpression networks in which genes are associated in the context of other shared coexpressed genes; (2) Decomposition of the SCCM using a novel heuristic algorithm termed "Triple-Link", which searches the highest connectivity in the SCCM, and then uses two connected TF as a primer for growing a TF cluster with a number of linking criteria. We applied TF-Cluster to microarray data from human stem cells and *Arabidopsis *roots, and then demonstrated that many of the resulting TF clusters contain functionally coordinated TFs that, based on existing literature, accurately represent a biological process of interest.

**Conclusions:**

TF-Cluster can be used to identify a set of TFs controlling a biological process of interest from gene expression data. Its high accuracy in recognizing true positive TFs involved in a biological process makes it extremely valuable in building core GRNs controlling a biological process. The pipeline implemented in Perl can be installed in various platforms.

## Background

Identifying the TFs potentially involved in a biological process is critical to unveiling regulatory mechanisms. Examples of the importance of identifying a small list of potentially crucial transcription factors include reprogramming somatic cells to a pluripotent state [1,2], the transdifferentiation of cells via forced TF expression [3] and genetic engineering of plants for increased productivity and adaptability[4]. Except for TF-finder [5], there is currently no methods or software specifically tailored to identifying TFs from expression data. Although some very well-performing network construction methods, for instance, CLR [6], NIR[7] and ARACNE [8], can be used to identify TFs from expression data, these methods are strictly TF-target oriented and output a well-connected regulatory network. Given that microarray data only measure a small component of the interacting variables in a genetic regulatory network[9] and that some portions of the nonlinear relationships between TF-targets are difficult to simulate and predict [10,11], identifying via TF-target modeling a short list of crucial TFs controlling biological processes in either mammals and plants is inefficient. As prior knowledge of target genes often do not exist, there is a need to develop new approaches for recognizing a short list of TFs controlling a biological process

With few sequence features among TF family that can be used to infer the functions of TFs, effective methods for identifying TFs that control a biological process have to rely on gene expression data or other datasets. Due to the challenges in generating time-series data with small intervals for higher plants and mammalian models, developing new methods that are applicable to compendium data sets pooled from multiple microarray experiments or public data resources is very useful. In this study, we collected microarray gene expression data from the same tissue types under similar conditions from multiple experiments to facilitate method development.

Genome-wide microarray data have shown that the coordination of functionally associated TFs is very noisy. This is because transcription is very complicated, with at least several TFs involved in establishing the transcriptional activity of any particular gene. An early study showed that transcription noise is partly due to a combination of variability in upstream signaling [12]. In addition, transcription for a particular gene can occur in bursts and can fluctuate, sometimes (but not always) in synchrony with biological processes such as the cell cycle [13] somitogenesis [14], or slow transitions between promoter states [12]. The abundance of TFs for a given gene or the number of transcription-factor binding sites within its promoter or enhancer can affect the amplitude, periodicity, and duration of transcriptional bursts [15]. In addition, the nucleosome positions and activities of chromatin remodelers can also cause transcriptional perturbation by the interconversion of a promoter between active and inactive states [16,17]. Moreover, chromatin domains also contribute to transcriptional variability; a change in the chromosome position of a gene affects not only its expression level but also its noisiness [18]. It has been shown that multiple copies of a given gene exhibit coordinated bursting when integrated in tandem, but exhibit uncorrelated responses when integrated at different chromosomal positions [19]. Noise in gene expression can disturb or impair the correlation and thus make the identification of coordinated TFs more challenging. In this regard, we should not anticipate that the TFs functioning in coordination have a perfect correlation or coordination and the mathematical methods that emphasize approximate "correlations" may recognize the functionally coordinated TFs more efficiently.

In this study, we developed a novel approach for identifying TFs involved in a biological process by building a conceptually new coexpression network represented by SCCM and then decomposing it into multiple subnetworks (or subgraphs) using Triple-Link, a heuristic algorithm that works as follows: it first searches all connected node pairs (genes) in the SCCM, and identify the one with highest connectivity, which is used as a primer for growing into a TF cluster. All TFs that are subsequently joined in need to have at least three significant connectivities to the TFs already in the cluster, with the exception of the third TFs that is required to have two. The cluster stops growing until there are no more nodes (TFs) meeting the requirement. A TF cluster is then produced. All TFs in this cluster are removed from the TF pool and SCCM matrix, and they do not participate in the next round of analysis. This process is repeatedly executed until all TFs are placed into clusters. The SCCM can be broken down into many subnetwork graphs because it is sparse and symmetric with both dimensions containing the same set of TFs. For such a graph, a few other graph clustering methods, including Markov Cluster Algorithm (MCL) [20] and affinity propagation (AP) [21], can also be applied to decompose it into multiple subgraphs. However, these methods were not developed specifically for decomposing the coexpression network we built in this study and thus may not produce outputs optimal for biological interpretation. In contrast to our other method TF-Finder [5], TF-Cluster does not require the use of any existing knowledgebase. We applied TF-Cluster to the microarray data from human embryonic stem cells during a transition from the undifferentiated ES state to a variety of differentiated states, and also applied to microarray data from Arabidopsis roots under salt stress. TF-Cluster recovers non-overlapping clusters containing important TFs recently identified as involved in controlling the pluripotency of human embryonic stem (hES) cells, human neural development and multi-directional differentiation, as well as Arabidopsis root growth and development in response to salt stress. In this study, functionally coordinated TFs refer to a group of TFs that are loosely coordinated in expression profiles as measured by the number of shared coexpressed genes. We postulate that these TFs control multiple facets of a biological process independently or combinatorially by controlling a set of target genes that may function in various subcellular components, in different cell types, or even in different organs if only they can function coordinately in time. As a result, the identified TF in the same cluster may not bind to the same group of target genes though it is very likely that a subset of TFs may bind to a subset of targets.

## Results

Using the pipeline containing Spearman rank correlation, the coexpression analysis was applied to both human and *Arabidopsis *data sets, and a SCCM was built for human and *Arabidopsis *respectively. We then decomposed the SCCM into subgraphs (clusters) with the Triple-Link algorithm. Since TF clustering was always initiated with a pair of TFs with maximal connectivity, a significant and also well-coordinated cluster is usually extracted earlier than a less significant one. To demonstrate the efficiency of this package and the Triple-Link algorithm, we examined some TF clusters derived from human and *Arabidopsis *data.

### Identification of functionally coordinated TFs during differentiation of human embryonic stem cells

As described in Methods, the microarray data set for human stem cells was collected from 17 experiments in which hES cells were treated with various differentiation reagents. Therefore, these datasets include states involved in many regulatory events underpinning pluripotency, such as ES maintenance, exiting the pluripotent state, and differentiation. If TF-Cluster is adequately efficient, we anticipated that those TFs involved in pluripotency would be identified at an early stage of execution. In fact, the first cluster identified was the one containing many known pluripotency-controlling transcription factors (see Table 1).

**Table 1:** TF cluster identified with pluripotency of human embryonic stem cells

Genes	Symbol	Description	Evidence
**Cluster 1: TFs control pluripotency renewal of human stem cells**			

NM_024865	NANOG	Nanog homeobox	[96]

BC099704	NANOGP8	Nanog homeobox pseudogene 8	Pseudogene with similarity to Nanog.

NM_003106	SOX2	SRY box 2	[96]

NM_002701	POU5F1	POU class 5 homeobox 1	[96]

NM_006892	DNMT3B	DNS methyltransferase 3 beta	[26,97]

NM_004078	CSRP1	cysteine-rich protein	

NM_080618	CTCFL	CCCTC-binding factor (zinc finger protein)-like	[98]

NM_016089	ZNF589	Zinc finger 589	[25,32]

NM_004426	PHC1	Polyhomeotic homolog 1	[22]

NM_005407	SALL2	SAL2 like	[25]

NM_018645	HES6	Hairy and enhancer of split 6	

NM_173547	TRIM65	Tripartite motif containing 65	

NM_004427	PHC2	Polyhomeotic homolog 2	[99]

NM_032805	ZFP206	Zinc finger protein 206 (ZSCAN10)	[23,24]

NM_001421	ELF4	ETS domain TF	

NM_003325	HIRA	HIR Histone Cell Cycle regulator	[100]

NM_033204	ZNF101	Zinc finger protein 101	

BC098403	ETV1	ETS variant 1	[28,29]

NM_006079	CITED2	Cbp/p300-interacting transactivator	[30,31]

NM_021728	OTX2	Orthodenticle homeobox 2	

NM_024015	HOXB4	Homeobox B4	

NM_006074	TRIM22	Tripartite motif-containing 22	[32]

XM_929986	LOC653441	Similar to polyhomeotic 1-like	Gene with sequence similarity to PHC1

NM_004497	FOXA3	Forkhead box 3	

**Cluster 22: TFs control neural development in earlier differentiation of human stem cells**			

BC008687	NEUROG1	Neurogenin 1	[101]

NM_006161	NEUROG1	Neurogenin 1	[101]

NM_001965	EGR4	Early growth response	

NM_033178	DUX4	Double homeobox 4	[38]

NM_006732	FOSB	FBJ oncogene homolog B	[35]

NM_003317	TITF1	NK2 homeobox 1	[39]

NM_002478	MYOD1	myogenic differentiation 1	[43,44]

NM_006192	PAX1	Paired box 1	[36]

NM_002700	POU4F3	POU class 4 homeobox 3	[37]

BC10493	POU4F3	POU class 4 homeobox 3	[37]

**Cluster 17: TFs control differentiation towards multiple directions in human stem cells**			

NM_001002295	GATA3	GATA binding protein 3	Trophectoderm [45]

NM_012258	HEY1	Hairy/enhancer-of-split related with YRPW motif 1	Trophectoderm [46]

NM_001804	CDX1	Caudal type homeobox 1	

NM_001430	EPAS1	Endothelial PAS domain protein 1	

NM_032638	GATA2	GATA binding protein 2	Trophectoderm [47]

NM_030379	GLI2	GLI family zinc finger 2	Mesoderm [48]

NM_017410	HOXC13	Homeobox C13	Ectoderm[51]

NM_002202	ISL1	ISL LIM homeobox 1	Mesoderm [49]

NM_033343	LHX4	LIM homeobox 4	

NM_002315	LMO1	LIM domain only 1 (rhombotin 1)	

NM_005461	MAFB	v-maf musculoaponeurotic fibrosarcoma oncogene Homolog B (avian)	Neural [53]

NM_002448	MSX1	Msh homeobox 1	

NM_002449	MSX2	Msh homeobox 2	Mesoderm[50]

NM_175747	OLIG3	Oligodendrocyte transcription factor 3	Neural [54]

NM_006099	PIAS3	Protein inhibitor of activated STAT, 3	Neural [55]

NM_019854	PRMT8	Protein arginine methyltransferase 8	Neural [56]

NM_030567	PRR7	Proline rich 7 (synaptic)	[102]

BC071571	RFX2	Regulatory factor X, 2 (influences HLA class II expression)	

NM_003068	SNAI2	Snail homolog 2 (Drosophila)	Neural Crest[57]

NM_031439	SOX7	SRY (sex determining region Y)-box 7	Endoderm (Parietal) [52]

NM_003150	STAT3	Signal transducer and activator of transcription 3 (acute-phase response factor)	

NM_003221	TFAP2B	Transcription factor AP-2 beta (activating enhancer binding protein 2 beta)	

NM_016267	VGLL1	Vestigial like 1 (Drosophila)	

NM_007129	ZIC2	Zic family member 2 (odd-paired homolog, Drosophila)	Neural [103]

NM_152320	ZNF641	zinc finger protein 641	

#### 1. TF cluster indentified with pluripotency of human embryonic stem cells

To demonstrate that this cluster is strongly correlated with human embryonic stem cell pluripotency, we examined each gene and the literature support for its involvement with pluripotency. PHC1 is implicated in pluripotency because its expression is repressed with the master pluripotency genes, OCT4 and NANOG, upon differentiation with retinoic acid (RA) [22]. ZFP206 (ZSCAN10) is a TF that controls pluripotency of embryonic stem cells by activating transcription of the OCT4 and NANOG promoters [23,24]. ZNF589, DNMT3A/B and SALL2 have been defined as pluripotency associated factors [25]. A novel DNMT3B splice variant was found to be expressed in pluripotent and cancer cells [26]. ES cells lacking the nucleosome assembly factor HIRA exhibit elevated levels of unbound histones, and the formation of embryoid bodies is accelerated, indicative of the onset of differentiation [27]. Embryoid bodies are aggregates of cells derived from embryonic stem cells. Upon aggregation, differentiation is initiated and the cells begin to recapitulate embryonic development to a limited extent. ETV1 is a direct target of NANOG and OCT4 in ES cells [28,29]. CITED2, as a TF playing key roles in mouse embryonic development, is involved in self-renewal and prevents spontaneous differentiation of E14Tg2a mouse ESC [30]. In addition, CITED2 is an essential regulator in adult hematopoietic stem cells [31]. Although their roles in ES cells are not clearly defined, *TRIM22 *and *ZIC3 *are believed to play a role in ES cells and have been used as ES markers [32].

This suggests that the TF-Cluster method is viable and can easily identify many of the key TFs reported in the literature as controlling the pluripotency of human stem cells. Of the 24 TFs in this cluster, 16 (~67%) have literature support for either being directly involved in the ES network or associated with ES cells. In the case of NANOGP8 and LOC653441, the literature contains evidence of a potential cross-hybridization with probes for known pluripotency regulators NANOG and PHC1 respectively. Although the other eight TFs - CSRP1, HES6, TRIM65, OTX2, FOXA3, ELF4, HOXB4, and ZNF101 - do not currently have supporting evidence, this does not indicate that they are not involved in pluripotency. For instance, HOXB4 has been indicated to play a role in the renewal of hematopoietic stem cells [33,34]. We believe future research will provide more clues regarding these particular genes. Nevertheless, our rediscovery of many important TFs involved in pluripotency maintenance using TF-Cluster suggests that it is highly efficient.

Cluster 1 contains three master TFs: Nanog, Sox2, and Oct4, which can bind to 1,330 active genes in stem cell independently or combinatorially. Among the 24 TFs of this cluster, only TRIM22 is bound by these three master TFs as indicated by the CHIP-on-chip data produced in previous study (Boyer, 2005). The same data also indicated that SALL2 is bound by Nanog only but at a location around 6.3 kb upstream. Nothing else is bound by these three TFs, suggesting the dominance of cooperation and synergy among the genes in a TF cluster. In addition, among these 1,330 active genes, 180 genes are controlled by these three master TFs, indicating that combinatorial control is not employed at a high rate (14%).

#### 2. TF cluster controlling neural development

Among the 189 human microarray data sets we used, about 60 were from early differentiation in which very earlier neural development can be tracked. We showed here that TF-Cluster can be used to identify the TFs controlling earlier neural differentiation. We simply searched a neural development marker, NEUROG1, which is contained in the 22^nd ^cluster. This cluster also contains several other genes involved in neural development (Table 1). Among these genes, NEUROG1 is involved in cortical neuronal differentiation. FOSB functions as a molecular switch underlying long-term neural plasticity [35]. *PAX1/E2A *double-mutant mice develop non-lethal neural tube defects that resemble human malformations [36]. Although the underlying mechanism is unclear, mutation of POU4F3 causes progressive hearing loss in humans [37]. DUX4 is highly expressed in embryonic neural tube by *in situ *hybridization [38]. TITF1 is implicated to play a role in the enteric nervous system [39]. MYOD1 is such a solid marker for muscle development [40-42] that its involvement in central neuron development in the brain is sometimes overlooked [43,44]. The literature support suggests that 90% (9 of 10) of the genes in this cluster are involved in neural development, indicating that TF-Cluster is capable of identifying clusters with a cohesive set of TFs that function in a biological process.

#### 3. TF cluster controlling differentiation towards multiple directions

The 189 human chips were collected from multiple experiments in which stem cells were treated with different reagents that triggered multiple types of differentiation. Usually the stem cells commit to differentiation at 48 hours upon treatment, and then enter a transition stage followed by further differentiation. We collected our data before 96 hours by which time early stages of differentiation, such as early neural differentiation, may be tracked, but more terminal differentiation to heart, brain, liver, kidney has not yet taken place. This early stage involves the formation of various lineage cells that are still in small quantity. All these various cell types, with no *a priori *knowledge, make it extremely challenging to interpret many clusters derived from this data set. We have shown the identification of the cluster involved in pluripotency renewal and the cluster involved in neural development. If we could identify a TF cluster controlling earlier differentiation towards multiple directions, it is an indication that the TF-Cluster pipeline is sensitive and efficient in identifying TFs from data in a chaotic stage. We examined the outputs and found Cluster 17 contains 24 genes, among which 15 TFs are marker genes for trophectoderm[45-47], mesoderm[48-50], ectoderm[51], endoderm[52] and neural [50,53-57] differentiation (Table 1), clearly indicating that the differentiation of these cell types, from which different organs will be derived later, is well coordinated.

### Identification of functionally coordinated TFs during salt stress response of *Arabidopsis *roots

The *Arabidopsis *data sets used in this study were from salt stress microarray experiments on Arabidopsis roots. The same data set was used earlier [5] for identifying the TF regulators that control root growth in response to salt stress. In this study, we were mainly interested in the TFs involved in root growth and abscisic acid (ABA, a hormone induced by salt/water stress) responsive TFs. Therefore, we selectively interpreted a few clusters produced by TF-Cluster. These include Cluster 1, 2,5, 7, and 19, and the genes contained in these clusters are shown in Table 2. The TFs in Cluster 1 seem to function in root hair development. LRL3, for instance, is involved in root hair development [58]. Constitutive expression of RSL4 programmed constitutive root growth, leading to the formation of very long root hairs [59]. RHD6 is involved in the early formation of root hairs from epidermal cells [60,61]. Overexpression of the counterpart of RAP2.11 of barley in *Arabidopsis *results in root growth tolerance to high salinity [62]. TINY encodes a member of the DREB subfamily A-4 of ERF/AP2 transcription factor family (TINY). The mutant of TINY has short roots[63]. The expression of this gene is induced by ethylene, and appears to stimulate cytokinin biosynthesis. Both affect root growth [63]. FRU mRNA is detected in the outer cell layers of the root and accumulates in response to iron deficiency [64,65]. In Cluster 1, 83% of the TFs are involved in root growth. The TFs in Cluster 2 are clearly dominated by these genes known to control the stem cells in root cap (Table 2). We successfully discovered a subset of TFs that coordinately control cap growth and maturation. They include BRN1[66], BRN2 [66], SMB [67], FEZ [67] TOM7 [68], PTL2 [69] and TCP20 [70], which were recently identified as functioning coordinately in the stem cell niche and periphery tissues in root caps. For instance, FEZ and SMB control the orientation of cell division plane in Arabidopsis root stem cells, where FEZ promotes periclinal, root cap-forming cell divisions while SMB repress stem cell-like divisions in the root cap daughter cells via negatively regulating FEZ activity. In predivision stem cells FEZ activates expression of its negative regulator, SMB, constituting a feedback loop for controlled switches in cell division planes[67]. Interestingly, these TFs' activities are in concert with the activity of IAA33. Although there is currently no evidence supporting the idea that IAA33 plays a major role in root cap growth, auxin is the major hormone controlling many aspects of root growth and development[71]. In Cluster 2, there are also a few TFs that are involved in lateral root development. We visualize this as a coordinated event that happened near the root cap. The TFs in Cluster 5 are mainly involved in second wall growth and vascular development. These include VND7 [72], VND4 [72], SND2 [73], ADOF2[74], AT1G68810, LBD18 [75], MYB46[76], MYB52[76], MYB103[76], MYB20[76], and MYB54[76]. Some of these TFs have recently been identified to function in a TF interactive subnetwork as evidenced by the cited references and the information therein. In this circumstance, 69% of the TFs in Cluster 5 are involved in the vascular development. The TFs in Cluster 7 mainly control cell cycle and root growth. For instance, AtXR6 [77], DEL3 [78], and HMG1/2 [79] are involved in cell cycle control and progression. Three growth factors that include AtGRF, 1, 2, and 3, were identified by TF-Cluster. These TFs control growth and morphology although their exact functions in root have not been characterized [80,81]. Ectopic expression of MNP causes growth retardation, aberrant cell division patterns, and loss of meristem activity [82]. Finally PS1 is involved in meiosis and mutation of this gene causes cellular diploidy [83]. For Cluster 7, 89% of the TFs are associated with cell cycle. Finally, Cluster 19 contains TFs that are involved in ABA signaling or response, an event incurred by water deprivation or salt stress. These TFs include GBF3 [84], ABF4 (Yoshida et al. 2010), ANAC019 [85], ATHB7 [86], ATHB12 [86], ABF3 (Yoshida et al. 2010), RD26[87], MYB102[88]. In this case, 47% of the TFs are associated with ABA signaling.

**Table 2:** Cluster 1, 2, 5, 7 and 19 identified from salt stress data of Arabidopsis roots containing root growth and development

Gene	Symbol	Description	Evidence
**Cluster 1: TFs control the root hair growth**			

AT5G58010	LRL3	Roothairless1	[58]

AT5G19790	RAP2.11	Ethylene response factor controlling root growth	[62]

AT1G27740	RSL4	Postmitotic cell growth in root-hair cells	[59]

AT1G66470	RHD6	Early root hair formation	[60,61]

AT5G25810	TINY	ERF/AP2 TF control cell expansion in root	[63]

AT2G28160	FRU	Regulates iron uptake responses in outer cells of root	[64,65]

**Cluster 2: TFs control root cap development (stem cells of roots)**			

AT1G33280	BRN1	BRN1, SMB control root cap maturation	[66]

AT4G10350	BRN2	BRN2, SMB control root cap maturation	[66]

AT1G79580	SMB	FEZ and SMB control root stem cells	[67]

AT5G39820	ANAC094	Apical meristem protein, function unknown	[59]

AT1G26870	FEZ	FEZ and SMB control root stem cells in cap	[67]

AT1G74500	TOM7	Embryonic root initiation	[68]

AT3G27010	TCP20	Postembryonic cell division in root	[70]

AT2G30340	LBD13	Expressed in cells at the adaxial base of lateral roots	[104]

AT2G40470	LBD15	Expressed in cells at the adaxial base of lateral roots	[104]

AT1G51190	PLT2	Control root stem cell activity near cap	[69]

AT1G66350	RGL1	Root epidermal differentiation	[105]

AT2G37260	TTG2	Differentiation of trichomes and root hairless cells	[106]

AT5G57420	IAA33	IAA is involved in root development	[107,108]

AT2G29060		scarecrow transcription factor family protein	

AT5G07580		DNA binding/transcription factor	

AT1G21340		Dof-type zinc finger DNA-binding protein	

AT1G75710		C2H2-like zinc finger protein	

AT1G77200		DREB subfamily A-4 of ERF/AP2 transcription factor	

**Cluster 5: TFs control root vascular development, second wall growth development**			

AT1G71930	VND7	Regulates xylem vessel formation	[72]

AT5G12870	MYB46	Target of SND1, control second wall biosynthesis	[76]

AT1G01780	LIM	LIM domain-containing protein	

AT1G12260	VND4	Switches for protoxylem and metaxylem vessel formation	[72]

AT1G17950	MYB52	Second wall growth	[109]

AT1G63910	MYB103	Second wall growth	[109]

AT1G66230	MYB20	Second wall growth	[109]

AT1G68810	bHLH	Root vascular initial	[110]

AT1G73410	MYB54	Second wall growth	[109]

AT2G39830	DAR2	DA-1 related, control organ size	[111]

AT2G45420	LBD18	Lateral root and tracheary element formation	[75]

AT3G21270	ADOF2	Early stages of vascular development	[74]

AT4G00220	JLO	A central regulator of auxin distribution and signaling in root	[112]

AT4G28500	SND2	Vascular cell differentiation	[73]

AT5G66610	DAR7	DA-1 related, control organ size	[111]

**Cluster 7: TFs control root cell cycle & growth**			

AT5G24330	AtXR6	Cell cycle regulation of late G1 to S phase	[77]

AT3G01330	DEL3	Cyclin D/retinoblastoma/E2F pathway	[78]

AT2G22840	AtGRF1	Growth factor expressed in root	[80,81]

AT2G36400	AtGRF3	Growth factor expressed in root	[80,81]

AT4G37740	AtGRF2	Growth factor expressed in root	[80,81]

AT3G50870	MNP	GATA transcription factor	[113]

AT1G34355	PS1	Parallel spindle 1 involved in meiosis	[83]

AT4G23800	HMG1/HMG2	High mobile group 1, 2	[79]

AT5G25475		Transcription factor B3 family	

**TFs control drought stress in response to ABA**			

AT2G46270	GBF3	induced by ABA under water deprivation	[84]

AT3G19290	ABF4	Regulate ABRE-dependent ABA signaling involved in drought stress	[114]

AT1G21000	Zinc	zinc-binding family protein	

AT1G51140	bHLH	Drought stress	[115]

AT1G52890	ANAC019	Bind to drought-responsive cis-element in response to ABA	[85,87]

AT1G73730	EIL3	Ethylene signaling	[116]

AT2G18550	HB-2	DNA binding/transcription factor	

AT2G46680	ATHB7	Growth regulator in response to ABA	[86]

AT3G12980	HAC5	H3/H4 histone acetyltransferase/histone acetyltransferase	

AT3G61890	ATHB12	Growth regulator in response to ABA	[86]

AT4G21440	MYB102	ABA-induced protein	[88]

AT4G25480	DREB1A	Drought stress genes responsive to ABA	

AT4G27410	RD26	Transcriptional activator in ABA-mediated dehydration response	[87]

AT4G34000	ABF3	Regulate ABRE-dependent ABA-mediated dehydration response	[114]

AT4G37180	MYB	myb family transcription factor	

AT5G04760	MYB	myb family transcription factor	

AT5G47640	NF-YB2	NF-YB2 (NUCLEAR FACTOR Y, SUBUNIT B2); transcription factor	

### The efficiency of Triple-Link in decomposing SCCM network

Compared to existing graph methods, Markov cluster (MCL) algorithm [20] and affinity propagation (AP) [21], Triple-Link can decompose the SCCM more efficiently and results in biologically interpretable TF clusters. This is demonstrated by the functionally cohesive clusters shown in Table 1 and 2. The clusters resulting from MCL are usually bigger and often contain the genes in the clusters identified by Triple-Link (Table 3). For the two clusters with a size of 6 and 9 identified by Triple-Link as controlling *Arabidopsis *root growth, MCI identified two clusters with a size of 28 and 14 respectively that are supersets of the TFs identified by Triple-Link (Table 3). For the cluster controlling human stem cell pluripotency (Table 1), MCL produced a cluster of 219 TFs (not shown) that again is a superset of all those TFs shown in Table 1. A predicted cluster of this size is usually not valuable for biologists as there are too many entries for experimental validation, and various reprogramming studies [2,89,90] have shown that only a moderate number of TFs are needed to reprogram somatic cells to a pluripotent state. Conversely, AP tends to produce smaller clusters than Triple-Link. For the TF cluster controlling pluripotency, AP produced a cluster of 12 members while Triple-Link produced a cluster of 24 members (Table 1). These 12 TFs include NM_001452-FOXF2, NM_002701-POU5F1, NM_004426-PHC1, NM_004427-PHC2 NM_004497-FOXA3, NM_004502-HOXB7, NM_006079-CITED2, NM_024865-NANOG NM_033204-ZNF101, NM_145238-ZNF31, NM_152629-GLIS3, and XM_929986-LOC653441, with the master pluripotency master regulator SOX2 being separated to a different cluster. Six of these 12 have prior literature support for being involved in ES cell maintenance. However, the lack of inclusion of SOX2 indicates that this cluster may be too restrictive as SOX2 is a well-known regulator of ES pluripotency. For the TF cluster controlling neural development, AP produced a cluster of eight TFs, two less than the one identified by Triple-Link (Table 2). These eight TFs are BC008687-NEUROG1, NM_001965-EGR4, NM_002478-MYOD1, NM_002700-POU4F3, NM_006161-NEUROG1, NM_006732-FOSB, NM_152568-FLJ25169, NM_173849-GSC. AP also divided the Arabidopsis root growth clusters (shown in Table 2) into multiple clusters (Table 3). We examined eight genes in cluster 118, and found that four of them do not have firm literature support for a role in root growth. These four genes are AT1G10610, AZF1-AT5G67450, WRKY35-AT2G34830, WRKY36-AT1G69810, and WRKY19-AT1G68150. There are six genes in cluster 191, and three genes out of these six, APTX -AT5G01310, SUVH5-AT2G35160 and Wrinkled1-AT3G54320, show evidence of being growth genes. Cluster 143 contains 8 genes and three of them, TUBBY 8 (AT1G16070), AT5G25475, and EBS(AT4G22140) are lacking literature support for being growth genes. These results suggest that AP tends to produce smaller subgraphs that do not have cohesive functions. All this evidence suggest that Triple-Link outperformed both MCL and AP in that it can produce more functionally interpretable TF clusters with a size ideal for either functional analysis or experimental validation.

**Table 3:** Comparison of Triple-Link with MCL and Affinity Propagation

AGI	Cluster ID (TL)	Cluster ID (MCL)	Cluster ID (AP)
**AT5G58010**	**1**	**14**	**28**

**AT5G19790**	**1**	**14**	**28**

**AT1G27740**	**1**	**14**	**28**

**AT5G25810**	**1**	**14**	**28**

**AT1G66470**	**1**	**14**	**118**

**AT2G28160**	**1**	**14**	**118**

**Cluster size**	**6**	**28 (Others not shown)**	**Size: Cluster 28: 5 TFs Cluster 118: 10 TFs**

**AT2G36400**	**7**	**15**	**140**

**AT3G01330**	**7**	**15**	**140**

**AT3G50870**	**7**	**15**	**140**

**AT4G37740**	**7**	**15**	**140**

**AT1G34355**	**7**	**15**	**143**

**AT4G23800**	**7**	**15**	**143**

**AT5G25475**	**7**	**15**	**143**

**AT2G22840**	**7**	**15**	**191**

**AT5G24330**	**7**	**15**	**191**

**Cluster size**	**9**	**14 (Others not shown)**	**Size: Cluster 140: 5 TFs Cluster 143: 8 TFs Cluster 191: 6 TFs**

**Figure 1:**
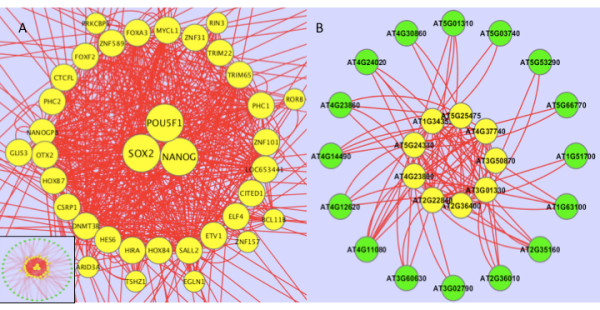
**The clusters identified by Triple-Link are well connected between any nodes within each cluster**. 1A: Cluster 1 (yellow nodes) contains the TFs predicted to be involved in pluripotency in human embryonic stem cells. The three genes in the center are master TFs, NANOG, POU5F1, and SOX2, which are crucial for pluripotency. A few nodes located immediately outside the inner ring are those that may not be always captured, depending on the parameters used. 1B: Cluster 7 (yellow nodes) contains the TFs controlling root growth in Arabidopsis under salt stress.

In addition to proving the efficiency of TF-Cluster by comparing with other methods, we also examined the number of connectivities within the derived clusters and between each cluster and other genomic genes. Two examples were shown in Figure 1A and 1B. It is obvious that the connectivities between TFs within the cluster are much more than those between TFs within a cluster and other genomic genes, suggesting that TF-Cluster can generate clusters by breaking down connected TFs from the weakest links.

Although we have demonstrated that Triple-Link performed better than AP and MCL in generating optimal cluster size, we have tested it to only two data sets. For this reason, we suggest users to try Triple-Link together with AP and MCL with multiple parameter choices so that it can be tested with a variety of data sets from various experimental conditions and species. What is interesting is that after we developed and tested Triple-Link, we found it can produce a cluster size that is between those that can be generated by AP and MCL. In this sense, AP, Triple-Link, and MCL form an array of methods for decomposing SCCM matrix. Although we believe Triple-Link performs better in decomposing SCCM because it was specifically designed and tuned up for this purpose, firm conclusion can be drawn only upon extensive tests being completed.

### Spearman rank correlation is a better method than Pearson correlation for associating TFs that have loose coordination

The success in identifying many TF clusters with functional coordination can be at least partially ascribed to the efficiency of the Triple-Link decomposition algorithm. To explore how the method used for measuring gene association can affect results, we compared Spearman rank correlation with Pearson product-moment, which is widely utilized in almost all coexpression analyses. Due to the noise arising from complicated interactions during transcription, we hypothesize that the Spearman rank correlation that emphasizes looser trend correlations may perform better in identifying functionally coordinated gene clusters, as shown in an early study[91]. To prove Spearman rank correlation is a better choice for the purpose of this study, we evaluated the coexpression analysis outcomes resulting from simple linear regression and Spearman and found that the Spearman's rank correlation indeed performs better than linear regression in finding clusters of biologically associated genes.

Spearman's rank correlation coefficient (called "rho") is a non-parametric (distribution-free) rank statistic [92], which is a measure of the strength of the association between two variables when the data are ordinal or do not follow a Gaussian distribution. It is a measure of a monotone association used when the distribution of the data makes Pearson's correlation coefficient undesirable or misleading. To test this, we performed the Shapiro-Wilk normality test and found that among 16,219 expressed genes, only 996 genes have a p value > 0.05, suggesting that the expression of most individual genes do not strictly follow a Gaussian distribution, further suggesting that the use of non-parametric methods may be more appropriate.

**Figure 2:**
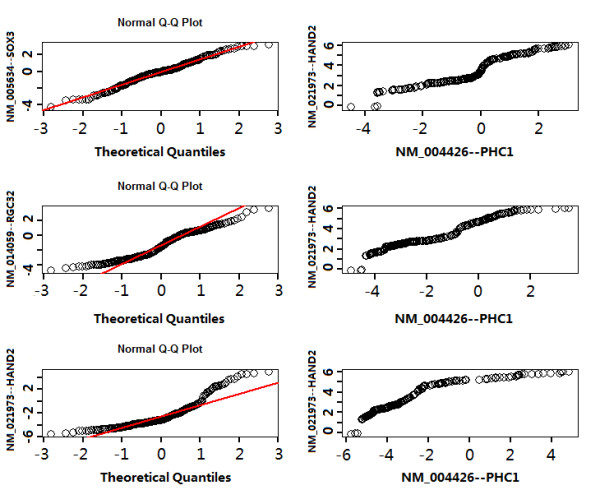
**Normal Q-Q plot and Q-Q plot of a few genes for which Spearman and Regression make no difference (SOX3), and for which there is a difference (RGC32 and HAND2)**.

To explain how the distribution of a gene influences its rank in the coexpressed gene lists when different association methods are employed, we used NM_004426-PHC1 as an example. We set PHC1 as a dependent variable and then examined the ranks of some other genes that have either a normal distribution or ones that depart from the normal distribution. This can be accomplished by a graphic method called Q-Q plot in which the quantiles of two variables are plotted again each other. These plots are displayed in Figure 2.

In Figure 2, we showed that SOX3 has an approximate normal distribution because the points in the normal Q-Q plot (top left) lie approximately in a straight line. In this case, SOX3 is the 47^th ^most coexpressed gene with PHC1 regardless of whether Spearman, Pearson, or regression is used. Q-Q plots shown in Figure 2 suggested that RGC32 and HAND2 deviate from the normal distribution. For these genes, we found that Spearman and regression/Pearson do make a difference. For the genes that deviate in a manner similar to RGC32 (where the observed highest quantiles are less than the highest theoretical quantiles), linear regression (LR, hereafter) gave a higher rank (41^st^) while Spearman gives a lower rank (86^th^) in the list of genes coexpressed with NM_004426 (PHC1). Several other genes including NM_002448--MSX1 (LR 42^th^, Spearman 65^th^), NM_005270--GLI2(LR 39^th^, Spearman 61^th^), NM_007129--ZIC2 (LR 27^th^, Spearman 48^th^), NM_012204--GTF3C4(LR 41^th^, Spearman 86^th^), NM_033132--ZIC5(LR 33^th ^Spearman 60^th^) showed this type of deviation in that all have a higher rank when regression/Pearson is used rather than Spearman. For the genes that deviate in a manner similar to HAND2 (where the observed highest quantiles are greater than the highest theoretical quantiles), Spearman gave a higher rank while linear regression/Pearson gave a lower rank (75^th^) in the coexpressed gene list with PHC1. Several other genes having this type of deviation include NM_005253--FOSL2 (LR 81^th^, Spearman 50th), NM_005257--GATA6 (LR 82^th^, Spearman 56^th^), NM_005342--HMGB3(LR 91^th^, Spearman 69 ^th^), NM_023033--METTL1(LR 95^th^, Spearman 58), NM_002653--PITX1(LR 72^th^, Spearman 31^th^). We chose PHC1 as the dependent variable in regression because it does not have a normal distribution itself (Figure 2, top, right panel) and thus can represent most other genes that do not obey a normal distribution.

Having demonstrated that Spearman and Pearson indeed have some differences in identifying coordinated TFs, we now show that Spearman is capable of capturing more biologically meaningful relationships with gene expression data. We examined the overlap of the top 50 most tightly coexpressed genes between three master TFs regulators, NANOG, POU5F1, and SOX2. When Spearman correlation was employed, we obtained 35 coexpressed genes that were common in three gene lists, each containing the top 50 genes most coexpressed to NANOG, POU5F1 and SOX2 (Table 4). When regression/Pearson correlation was used, we obtained only 24 common genes. Of the 35 identified by Spearman correlation analysis, 22 are common to the 24 genes identified by regression, and 13 are unique to Spearman. Eight out of these 13 genes have literature support for being involved in hES cells. This suggests that Spearman correlation is capable of capturing a larger list of functionally associated TFs, possibly because of its ability to capture those with a looser coordination in expression.

**Table 4:** The intersection of coexpressed genes to NANOG, SOX2, and POU5F1 when Spearman and regression are used

Common Genes	Unique Genes
**BC069807--NANOGP8**	Regression/Pearson
**BC090958--SALL2**	
**BC099704--NANOGP8**	**BC093979--HESX1**[117]
**NM_001421--ELF4**	**NM_032805--ZNF206 (ZSCAN10) **[23,24]
**NM_003106--SOX2**	
**NM_003325--HIRA**	Spearman
**NM_004078--CSRP1**	**AF454056--PRKCBP1 **[60]
**NM_004426--PHC1**	**BC010105--NASP**
**NM_004427--PHC2**	**BC098403--ETV1 **[28,29]
**NM_004497--FOXA3**	**CR627389--ETV1 **[28,29]
**NM_005375--MYB**	**NM_002653--PITX1**
**NM_005634--SOX3**	**NM_002701--POU5F1 **[25]
**NM_006892--DNMT3B**	**NM_005224--ARID3A**
**NM_016089--ZNF589**	**NM_005239--ETS2 **[118]
**NM_018645--HES6**	**NM_005407--SALL2 **[25]
**NM_021728--OTX2**	**NM_006074--TRIM22 **[32]
**NM_022051--EGLN1**	**NM_021958--HLX1**
**NM_024504--PRDM14**	**NM_021973--HAND2**
**NM_024865--NANOG**	**NM_024015--HOXB4 **[33]
**NM_033204--ZNF101**	
**NM_173547--TRIM65**	
**XM_929986--LOC653441**	

## Discussion

### The SCCM is a more informative measure for TF coordination than simple coexpression

Existing coexpression analysis is typically either correlation- or regression-based. Due to the variation in the strength of coordination between TFs or TFs and other genes, mandatory implementation of any cut-off threshold in correlation or regression-based coexpression analyses often leads to the elimination of those TFs having relatively weak coordination strength with other genes. An example would be where TF *A *is involved in stress response and another TF *B *is a regulator controlling organ development. Due to the need for rapid response under stress conditions, TF *A *may have 100 coexpressed genes with a correlation coefficient varying between 0.85~0.95 while *B *has 100 coexpressed genes with a correlation coefficient varying from 0.55 ~0.70. Genome-wide coexpression analysis often disregards TF B due to its low coexpression strength with other genes. We argue that TFs with relatively lower coexpression strength may be intrinsic to the characteristics of some cellular activities or events and that this lower coexpression strength should not be used *a priori *to eliminate TFs. The SCCM was developed to deal with this issue so that TFs with lower association strength are not eliminated at an early stage. However, at the decomposition stage, any TF that does not share coexpressed genes with other TFs was automatically eliminated.

**Figure 3:**
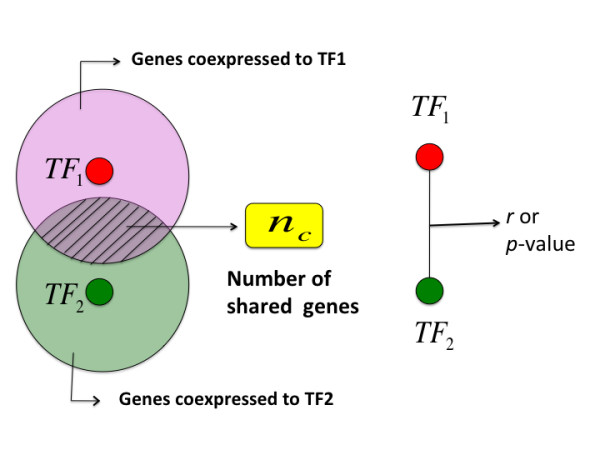
**Shared coexpression connectivity represents the coordination of two TFs in the context of other genes while correlation coefficient/regression p value between two TFs only reflects the distance between these two TFs with regard to coexpression**.

When the coordination between two TFs is measured by the number of coexpressed genes, the context of all genes genome-wide are taken into account (Figure 3). Therefore, every entry in SCCM reflects a more biologically meaningful measure as compared to the absolute distance represented by the correlation coefficient or regression p value.

### TF-Cluster identifies different TFs as compared to TF-finder

Interestingly, most TFs controlling root growth as identified by TF-Cluster are different from those identified by TF-finder [5]. For instance, TF-finder identified a B3 family TF (AT2G16210) and GRF7 while TF-Cluster identified GRF1, 2 and 3, and a different B3 family member (AT5G25475). This is not surprising because the two methods use different principles. TF-finder uses bait and guide genes aided by multiple correlation analysis to identify TFs while TF-Cluster uses relatively loose coordination analysis following by network decomposition. The TFs within each cluster identified by TF-Cluster are typically cohesive in function as shown in Table 1 and 2. Such a phenomenon was not observed in the outputs resulting from TF-finder [5]. Disparate functions of the genes shown in Table 1 and supplemental Tables in the TF-finder article [5] are obvious and such a feature is probably rooted in the use of bait and guide genes, which have distinct functions..

### Interpretation and validation of TF cluster function

To identify the function of each derived cluster, the user may need to search the existing recent literature. This is because, in the existing database, most genes encoding TFs have very simple annotation. For example, an annotation may read as follows: molecular function: transcription activity; cellulose component: nucleus; biological process: regulation of transcription. This kind of annotation does not help in figuring out the function of a TF-Cluster. When applied to higher plants and mammals that have a variety of cell types and developmental stages, caution needs to be exercised in interpreting the functions of each TF in a cluster. It is also important to find the articles whose research was done using the same tissue types. A cluster of TFs recognized from TF-Cluster contains those that are loosely coordinated in expression. There is thus no guarantee that these TFs function in the same cells, or bind to the same set of target genes though it is highly likely that a subset of them may share a subset of target genes. For example, the roots are comprised of many cell types, and several TF clusters we identified apparently function in different locations. For instance, Cluster 2 contains TFs that function at the tip of roots (Table 2), and Cluster 5 contains TFs that function in vascular tissues (Table 2). Although TFs in the same cluster can be involved in remote events in different cell types in the same organ or even whole plants, depending on how the samples are harvested, the confirmation of subset TFs binding to a set of target genes is still helpful in consolidating the cluster. In this regard, the availability of CHIP-seq or CHIP-on-chip data from the specific cell types will aid cluster validation. Although this kind of data is still scarce at the time being, the DREAM project has started to collect data to facilitate this kind of validation (http://wiki.c2b2.columbia.edu/dream/index.php/The_DREAM_Project). In addition, with the availability of third generation sequencing technology, this kind of data will soon become widely available. Currently, examination of co-existing cis-elements bound by the different TFs of the same cluster is helpful if the method is applied to data from bacteria and yeast. However, it is of little value if the method is applied to higher plants and mammals simply because we currently do not know to which motifs these TFs bind. Caution must be taken in using motif information to interpret the clusters because the mere presence of a motif does not indicate it is an active one. In this sense, CHIP-on-chip or CHIP-seq data are more valuable.

Although not absolutely required, a general understanding of the biological process of interest and also the data collected can help interpret the derived TF clusters. It is important to recognize the limitation of any particular data set and to avoid over-interpretation of the derived TF clusters. Generally speaking, the biological process of interest should be activated and dominant in the data collected. If one cannot identify a TF-cluster for a specific biological process, try to get adjacent spatial or temporal data sets. This will become possible when we have ample gene expression data in a public domain.

### How many coexpressed genes should be used to measure coexpression between two TFs when SCCM is constructed?

It is conceivable that the use of the top 50, 100, and 150 could not significantly affect true positive rate for each cluster. This is because these genes are used as a measurement, not as participants. To get an idea of which choice is optimal, we examined two median size clusters: human Cluster 1 and Arabidopsis Cluster 2 for cluster size and true positive discovery rate with respect to the different schemes of top genes, and obtained the following results:

For human Cluster 1, three schemes of top 50, 100 and 150 yielded three clusters of 22, 24 and 31 TFs, respectively, with positive rates of 77.2%, 66%, and 55%. For Arabidopsis Cluster 2, three schemes of top 50, 100 and 150 yielded three clusters of 14, 18 and 22 TFs, respectively, with positive rates of 71.4%, 72.2%, and 63.6%. These results indicate that the use of 50 is good but may have less prediction power for novel genes; that the top 150 could not only potentially increase the size of the cluster but also introduce false positives; and that the top 100 can achieve higher positive discovery rate than the top 150 while maintaining decent prediction power of novel TFs. Nevertheless, we suggest users compare the three schemes in real application because other factors like data size and genes involved in the biological process of interest can also affect the cluster size and accuracy.

## Conclusions

TF-Cluster can be used to cluster all TFs into multiple clusters of various sizes using gene expression data from a biological process. Each cluster contains the TFs assumed to function coordinately in time to regulate the multiple facets of a biological process. The TF-Cluster algorithm outputs the TF clusters according to the order of association. Clusters of closely associated TFs in the coexpression networks will be displayed earlier. Compared to TF-finder, TF-Cluster can identify many groups of TFs, each with a cohesive function. TF-Cluster does not require an existing knowledgebase, and thus can be used more widely if only the microarray data representing many "snapshots" of a biological process are available. With the increased availability of gene expression data in public resources, TF-Cluster will no doubt have a wide variety of applications in the future. Nevertheless, TF-Cluster may not be useful when a compendium data set contains no more than 30 samples/chips. This assumption is based on the fact that we have tried TF-finder to a data set containing 36 chips from Poplar, and we could identify TF clusters that can be explained biologically. In addition, TF-Cluster may not be applicable to some biological processes in which few TFs are involved and function with little overlapping in time. Finally, since the whole method is coexpression-based, the TF-Cluster pipeline can be potentially used for pathway analysis. It certainly can be used to identify coordinated or cross-talking pathways or predict new pathway genes. The pipeline was applied in Practical Extraction and Report Language (PERL), and parallel techniques were applied to accelerate the analysis (see Methods). For analyzing a data set comprised of human chips, and a coexpression network of 2,180 human TFs, it takes 2-4 hours in our Linux server. Interested users can send a request to Hairong Wei (hairong@mtu.edu).

## Methods

### Microarray Data and Data Preprocessing

#### Human microarray data set

One data set contains 104 high-density human gene expression arrays, each with 388,634 probes from 36,494 human locus identifiers from the HG17 assembly. These 104 chips were from 15 experiments in which stem cells were treated with different reagents that disrupted pluripotency while triggering differentiation; the reagents and the conditions included: TPA (a phorbyl ester) treatment in conditioned medium, TPA treatment in TeSR medium, BMP4 treatment with FGF, BMP4 treatment without FGF, and co-culture with mouse OP9 cells. The other dataset contains 85 high-density human gene expression arrays, each with 381,002 probes from 47,633 human locus identifiers from the HG18 assembly. This dataset was collected from a set of experiments where a variety of different growth factors were applied to human ES cells at varying conditions for 3 days. Both platforms were manufactured by NimbleGen Systems (http://www.nimblegen.com). All probes are 60 mers and all chips were hybridized to Cy5 labeled mRNAs extracted from human embryonic stem cells (hESCs) from undifferentiated to differentiated stages. Raw data were extracted using NimbleScan software v2.1. The two data sets were joined by gene mapping via selection of shared common probes between the same gene on the two platforms. More than 99.5% of mapped genes share more than 6 probes, and the signal intensities from these common probes were normalized with the Robust Multiple-chip Analysis (RMA) algorithm [93]. The whole dataset obtained contains 36,398 genes, which was used to construct coexpression matrix SCCM. Here we state that the stem cell research reported in this paper was approved under protocol SC02008-0002 of the Stem Cell Research Oversight (SCRO) Committee.

#### *Arabidopsis *microarray data set

Microarray data sets were downloaded from multiple resources. The salt stress experimental data set contains108 chips from 6 experiments (GSE7636, 7639, 7641, 7642, 8787, 5623) and was downloaded from the NCBI GEO website: http://www.ncbi.nlm.nih.gov/geo/. All data mentioned above are derived from hybridization of Affymetrix 25 k ATH1 microarrays [94]. The original CEL files were processed by the robust multiarray analysis (RMA)[93] algorithm using the Bioconductor package. For quality control we used methods that were previously described [95]. This data set was recently used for identifying TFs involved in salt stress response and growth[5].

### Building shared coexpression connectivity matrix (SCCM)

Let *T *= {*y*_1_, *y*_2_,...,*y*_*p*_} denote the set of TFs known in a genome, and *y*_*i *_(*i *= 1,...,*p,m *≥ *ψ*) = {*e*_*i*1_, *e*_*i*2_, *e*_*i*3_, ..., *e*_*im*_} is the gene expression profile of the *i^th^*TF in *m*^*th *^microarray chip. Also let *G *= {*x*_1_, *x*_2_,...,*x*_*q*_} represent all genes in the genome, and *x*_*q *_(*i *= 1,...,*n,m *≥ *ψ*) = {*e*_*q*1_, *e*_*q*2_, *e*_*q*3_, ..., *e*_*qm*_} is the gene expression profile of the *q*^th ^gene in *m*^*th *^microarray chip. For this analysis, *ψ *is the minimally required number of chips that should be used for this kind of analyses (our empirical *ψ *≥ 50). For each pair of *y*_*i*_and *x*_*q *_(*i *= 1,...*p*; *q *= 1,...,*n*), a Spearman rank correlation rho  or a regression p value is calculated (Persson et al., 2005; Wei et al., 2006). Where  and  are the ranks of TF *i *and TF *q*. Then for each *y*_*i*_, we rank all the genes in *G* by *ρ*_*iq *_and retain the top Ω ( Ω can be 50, 100, or 150) genes that are co-expressed most closely with *y*_*i*_, then denote this set of genes as *G*_*i*_.

**Figure 4:**
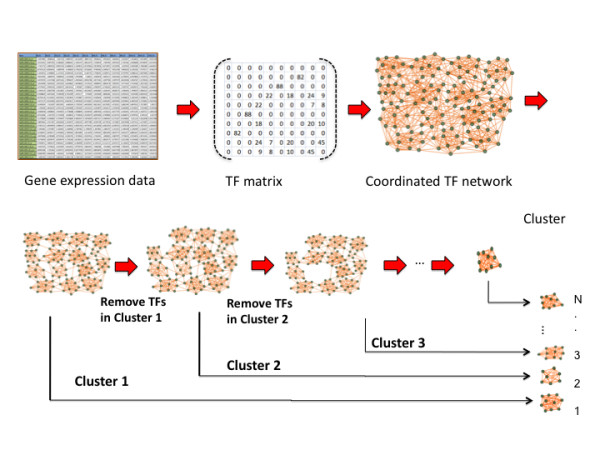
**The workflow of TF cluster**. Automated package that can recognize transcription regulators controlling a biological process with gene expression data (microarray or RNA-seq). The TF-recognition can be classified into two phases: construction of TF coexpression network and decomposition of the network into coordinated TF clusters. The package was developed with Perl, and thus can be used in various platforms.

After the above co-expression analysis, we are now able to build a *p *× *p *symmetric matrix *A*, whose both row and column variables are the TFs in *T *(Figure 4), and each entry *a*_*ij *_(*i, j *= 1,...,*p*) represents the number of shared most co-expressed genes between TFs *y*_*i *_and *y*_*j*_, that is the number of common genes between *G*_*i *_and *G*_*j*_, namely , Hereafter, we defined *a*_*ij *_as the number of connectivity (*n*_*c*_) for the pair of TFs.

### Decomposition of SCCM transcription factor network

Given matrix A and a set of TFs, *T *= {*y*_1_, *y*_2_, *y*_3_,...,*y*_*p*_}, we can now decompose SCCM for the clusters of TFs. The TFs in each cluster are assumed to work together to achieve some kind of functionality in a biological process. To achieve this, we developed a heuristic algorithm, Triple-Link, which always uses the two TFs with the maximal *n*_*c *_as a primer, and gradually adds other TFs that have significant connectivity with TFs that are already in the primer or primer-derived cluster. A significant connectivity is defined as the one with a value larger than a threshold of *n*_*c *_>*μ *+ *θδ*, where *μ *and *δ *are the mean and the standard deviation of non-zero connectivities contained in SCCM respectively. We have three *θ*_1_, *θ*_2_, *θ*_3 _that are corresponding to three thresholds that were used to determine if another candidates should be joined, with *θ*_1_, to be the most and *θ*_3 _to be the least stringent one. Our empirical values of three theta are located within the following ranges: *θ *= ({*θ*_1_,*θ*_2_,*θ*_3_} ⊂ ({2.5 ~ 1.5,2.0 ~ 1.0,1.5 ~ 0.5}), where *θ*_1_ >*θ*_2_ >*θ*_3 _is required for implementation of different stringency. Since each cluster started with two TFs, the third TF was added in on the condition that it had only two significant connectivities with the existing two TFs. After this, we required a candidate TF to have only three significant connectivities with any TFs that were already in the cluster grown from the primer regardless of the size of existing cluster. Once a candidate TF was included in the cluster, it was then removed from T. This process was then repeatedly executed until there were no more candidate TFs that shared at least three significant connectivities with the TFs within the cluster. All TFs in the cluster were removed from. *T *= {*y*_1_, *y*_2_, *y*_3_,...,*y*_*p*_}. This process was then repeatedly executed until all TFs in *T *= {*y*_1_, *y*_2_, *y*_3_,...,*y*_*p*_} with significant connectivity were removed. The detail procedure of this algorithm is described below:

A workflow for TF pipeline is shown in Figure 4.

### Acceleration of TF-Cluster pipeline by enhancing CPU usage and eliminating non-essential steps

Genome-wide coexpression analysis of all TFs, building SCCM, and decomposition of SCCM are all computationally intensive. To reduce computing time, we implemented multiple techniques to shorten the running time. The measures we took included: (1) Using Perl rather than R. Correlation matrix building is one of the most time consuming steps of this pipeline. For example, we needed to perform 2180(TFs) x 16219 (genes) = 35,357,420 correlation analyses with the human data of 189-dimentional samples, which took 10 days to run in R. When we switched to Perl, we could complete above-mentioned correlation analyses in 1-2 hours; (2) Introducing parallel computing. Almost all computational servers now have multi-core processors that allow for parallel computing. To take advantage of this, we parallelized all non-sequential parts of the code, which increased the speed by a factor of at least 3 on our server. (3). Using a better algorithm. The loop is the most expensive operation in any computing language. We removed several loops by index point checking. (4) Reducing unnecessary Input/Output (IO). IO can significantly impact computational speed. Just the correlation matrix file alone produced in this study exceeds 3 GB. To avoid unnecessary IO, we processed a single TF against all other genes' correlation on the fly, and only the top correlated gene list was output. This step reduced the memory usage by factor of 1000. (5) Avoid functions and modules if possible. Every function and module consumes over 1kb for its mere existence. Function and module loading as well as argument passing take a lot of computational resources. We optimized code into only two concise scripts by replacing a lot of modules with only few lines of code, which made the program not only more efficient but also portable. For analyzing a data set comprising of human chips, and a coexpression network of 2180 human TFs, it took 2-4 hours in our Linux server (Dell PowerEdge Server 2990 III with Intel Xeon X5640 quadcore processor (3.16ghz), and 48 GB RAM).

## Availability and requirements

The TF-Cluster pipeline was written in Perl. We will release the executable files free to academic, but may charge license fee for any commercial uses. Original source codes of the software can be made available under a suitable open-source agreement. For details, please contact: hairong@mtu.edu.

## Authors' contributions

JN developed the Perl code for building the SCCM matrix, merged the pipeline, and tuned-up the running time. RS was involved in project design, analysis of clusters, and writing part of the manuscript. HZ was involved in network plots, and code testing, FR ran AP and MCL, XC, contributed to the method. JT contributed human microarray data set and involved in manuscript edition. HW merged and preprocessed human data from different platforms, developed the overall frame of this project, Triple-Link algorithm and Perl code for Triple-Link, and wrote the majority of manuscript. All authors read and approved the final manuscript.
